# First Report of Hereditary Lysozyme Amyloidosis in a South Asian Family

**DOI:** 10.1155/2019/5092496

**Published:** 2019-02-10

**Authors:** Madiha Iqbal, Prachi Jani, Salman Ahmed, Taimur Sher

**Affiliations:** Department of Hematology and Oncology, Mayo Clinic, Jacksonville, FL, USA

## Abstract

Lysozyme amyloidosis (ALys) is an exceedingly rare autosomal dominant hereditary type of systemic amyloidosis that can be misdiagnosed as other common types of systemic amyloidosis. The gastrointestinal tract and the kidney are the most common sites of organ involvement. No specific treatment exists for ALys, and the management primarily consists of organ-directed supportive care. To our knowledge, this disorder has been previously reported only in European ancestries; here, we first report the occurrence of ALys in South Asian ancestry. This report highlights the need of awareness amongst physicians regarding the extension of this unique and challenging disorder to non-European ancestries.

## 1. Introduction

Amyloidosis is a fascinating group of disorders characterized by extracellular deposition of amyloid fibrils in tissues and organs. Amyloid fibrils are derived from misfolded proteins in beta-pleated sheets from a wide variety of protein precursors (over 30 precursor types known) that can cause diseases belonging to a wide spectrum of human disorders such as familial cardiomyopathy (transthyretin amyloidosis), neoplastic disorders of plasma cells (immunoglobulin light chain amyloidosis), neurodegenerative diseases (Alzheimer's disease), and infectious diseases (prion disease) among others. This systemic amyloidosis can be acquired or hereditary depending upon the type of protein and the site of mutation (germline vs. acquired). Lysozyme amyloidosis, noted as ALys in the latest amyloidosis nomenclature, is an extremely rare nonneuropathic hereditary type of amyloidosis. In its normal form, lysozyme, as part of the innate immune system, plays an important role in mucosal defenses at sites such as the mucosal lining of the aerodigestive tract and conjunctiva. In ALys-specific germline, mutation(s) render the mutant lysozyme prone to misfolding in beta-pleated configuration giving rise to lysozyme amyloidosis. Only a handful of mutations have been reported to cause ALys, and these predominantly affect the gastrointestinal tract, kidney, and lymph nodes. A high degree of penetrance is noted in affected families; however, clinical presentation is quite heterogeneous among affected family members. To the best of our knowledge, all families that have been reported in the literature belong to the European ancestry (Table 1). Here, we describe a novel mutation causing ALys in a South Asian pedigree.

## 2. Case Presentation

The proband is a 39-year-old South Asian female of Indian origin who was diagnosed with systemic amyloidosis of unknown type when she was 16 years of age. At the time of diagnosis, she was pregnant and was experiencing gastrointestinal symptoms of abdominal bloating, dyspepsia, heartburn, and nausea. These symptoms persisted postpartum, and the patient underwent an upper endoscopy with biopsy, which was consistent with the presence of amyloid material; however, the type could not be determined. Thereafter, the patient continued to live a fairly normal life and did not seek any further medical attention until her daughter who when turned 16 started experiencing similar symptoms and underwent an upper endoscopy with biopsy results consistent with the presence of amyloid. At that time, the family sought medical attention at our institute. The proband reported that, in spite of an overall normal life, she had continued to experience dyspeptic symptoms that worsened with stress. She also reported around 12–15 episodes of hematemesis since her initial diagnosis. Additional symptoms on close questioning included easy bruisability, periorbital purpura, and ecchymosis associated with activities such as retching and vomiting. An upper endoscopy was performed at the Mayo Clinic. No morphological abnormality was seen on the endoscopy; however, Congo red stain demonstrated the presence of amyloid material in the gastroesophageal junction, stomach, and duodenum. Serum and urine protein electrophoresis were normal. Proteomic assessment with laser capture mass spectrometry (LCMS) evaluation of the congophilic material detected an amino acid sequence abnormality in the lysozyme protein (I56T). Peripheral blood genotyping confirmed I56T mutation (DNA change c.221T > C) in exon 2 of the lysozyme gene, which replacing isoleucine with threonine at position 56. I56T mutation per human genome variation society (HGVS) nomenclature is now known as I74T. Patient's renal and hepatic functions were normal, and she did not have any adenopathy. Besides iron deficiency and mildly delayed gastric emptying, no other abnormality was noted. The patient continues with yearly follow-up in our amyloidosis clinic with periodic exacerbations of dyspeptic symptoms and a few episodes of small-volume hematemesis, which have responded to high-dose acid suppressive treatment.

## 3. Family History

Proband's daughter at 16 year of age started experiencing anxiety and upper abdominal pain along with dyspeptic symptoms, which lead to an upper GI tract endoscopy and biopsy confirming I56T mutant Alys via LCMS. Her most significant symptoms were indigestion, anorexia, altered bowel habits, and easy bruisability. She was also noted to have moderate iron deficiency anemia secondary to heavy menstruation. Her symptoms subsequently improved with change in dietary habits. At the time of most recent evaluation, which was a year ago, the patient has been doing relatively well with the exception of intermittent nausea and abdominal bloating.

Proband's only other child is a 12-year-old son who diagnosed with “Henoch–Schonlein Purpura (HSP)” at the age of ten. He is currently doing well, is without any symptoms, and is in the process of genetic counseling.

Proband's extended family is settled in India; however, various members of the family are suspected to be suffering from symptoms that are likely secondary to lysozyme amyloidosis. All the affected members belong to proband's paternal side of the family without any report of any member suspected to be affected from proband's maternal side.

Proband's father who expired at age 67 secondary to cardiac valvulopathy is suspected of having suffered from lysozyme amyloidosis due to the report of prolonged hemorrhage after a sports incident as well as report of hematemesis that occurred on a few instances without any clear etiology after evaluation. Proband's brother who is currently 39 years of age suffers from frequent bouts of gastritis and subconjunctival hemorrhage and is considered possibly affected. Proband's father had a total of six siblings including him with three sisters and two brothers.

Proband's eldest uncle is currently alive and in his eighties who is reported to be healthy and does not have any offsprings. Proband's second uncle is in his sixties and suffers from kidney dysfunction without any clear etiology explaining the dysfunction and is therefore considered possibly affected in our report. He has recently started undergoing hemodialysis. He has two female offsprings with one of them undergoing a renal transplant in 2009. She was diagnosed with renal failure in her teens and was on hemodialysis for a few years before her transplant. She is now doing well. No clear etiology has been found for her kidney dysfunction after extensive evaluation and is therefore considered possibly affected. The other female offspring is reported to be healthy.

One of the paternal aunts passed away in 1994 after childbirth. She was in her 30s then and underwent a caesarean section; however, postoperatively, she had massive internal hemorrhage from her liver, which could not be explained by the local physicians. She is also reported to have suffered from bruising and gastritis prior to her death. She is considered as possibly affected.

Another aunt suffers from early stages of kidney dysfunction with reports of proteinuria. She was also evaluated as a possible kidney donor for her only daughter who is 30 years old and suffers from progressively worsening renal disease with significant amount of proteinuria and is currently being evaluated for renal transplant. However, as a part of evaluation as a potential kidney donor, a renal biopsy was done which was positive for the presence of amyloid deposition. Proband's cousin also suffers from frequent bouts of abdominal discomfort and dyspepsia.

**Figure 1:**
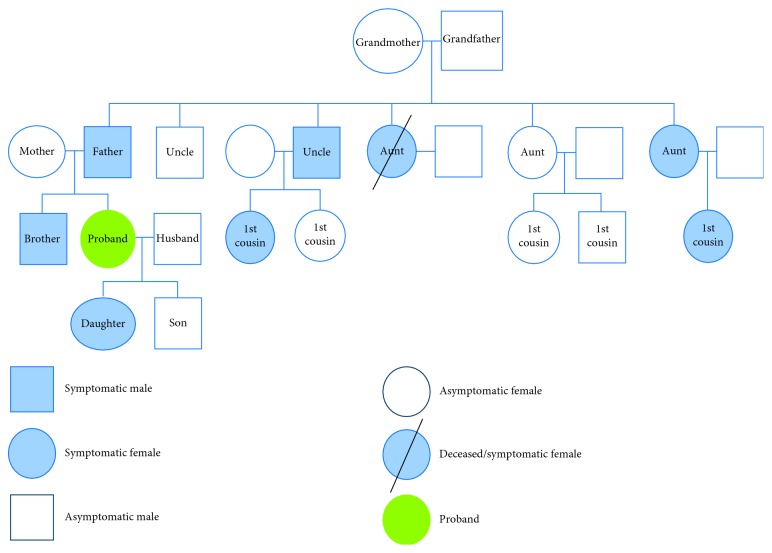
Family tree illustrating proband's paternal pedigree.

A family tree of paternal pedigree is illustrated in Figure 1.

## 4. Discussion

Lysozyme amyloidosis has been reported in a handful of families belonging to the European ancestry [4, 9, 11, 12]. There are no reports of ALys in the South Asian ancestry. Our report is unique in being the only report of an affected family of the South Asian origin.

The diagnosis of ALys continues to be challenging due to the rarity of the disease and nonspecific presentation with regard to symptomology in most instances. In a study of 350 patients with systemic amyloidosis who had been given the presumptive diagnosis of AL (light chain) amyloidosis, 34 patients (10%) were found to have hereditary amyloidosis after undergoing testing for amyloidogenic mutations highlighting the importance of including hereditary types of amyloidosis in the differential of systemic amyloidosis. In this study, one patient was found to have ALys [14].

While suspecting a diagnosis of hereditary systemic amyloidosis, a through family history is of paramount importance. Various methods are employed to diagnose lysozyme amyloidosis with the most common being the histological analysis of the lysozyme protein via immunohistochemistry. Other methods, which are more specific compared to histological analysis, include testing for DNA mutations and conducting laser microdissection with mass spectrometry.

The association of mutations in the lysozyme gene with systemic amyloidosis was first described by Pepys et al. in 1993 [15]. Unlike familial transthyretin amyloidosis (which is the most common form of hereditary systemic amyloidosis) where more than 100 pathogenic mutations are known, only a handful of amyloidogenic mutations have been reported in the lysozyme gene (W64R, I56T, D67H, and F57I) [4, 15]. Recently, another mutation in exon 2 caused by the substitution of T by A leading to replacement of Tyr by Asn (pTyr54Asn) has also been reported in a family with Swedish ancestry [9].

A heterogeneous presentation is quite notable among members of the affected family with the gastrointestinal tract being most commonly affected [11, 12]. Symptoms attributable to gastrointestinal (GI) involvement can be quite varied ranging from mild abdominal discomfort, nausea, to bleeding from the GI tract [11, 12]. Renal involvement is also quite frequently encountered, and varied degrees of renal impairment are seen ranging from mild dysfunction to the need of hemodialysis. W64R and F571 mutations have been noted to be associated with renal dysfunction [1]. Hepatic involvement leading to spontaneous hepatic rupture has also been reported though defined coagulation abnormalities like factor X deficiency in AL amyloidosis have not been reported in lysozyme amyloidosis [13]. Other organs that have been reported in the literature to be involved by lysozyme amyloidosis include salivary glands [16], lymph nodes [5], spleen [5], heart [10], and the bone marrow [2]. There does not appear to be direct corelation between mutation type and symptomology; however, most families present with involvement of a predominant single organ system. Our reported family however uniquely presented with involvement of multiple organ systems in various members including renal, gastrointestinal, and a propensity towards hemorrhage. Interestingly, a bleeding diathesis has been mostly seen in families harboring the D67H mutation; whereas our family carried the I56T mutation, again illustrating the observation that mechanisms other than the mutation type are behind various clinical presentations.

Currently, there is no specific treatment that is available for this rare disease. The median survival rate for ALys has been reported to be 17.9 years from diagnosis [17]. Treatment options currently are focused on alleviating patient symptoms. Kidney transplantation can be considered in patients who suffer from progressive renal failure. In a series of 104 patients who underwent renal transplant for various types of systemic amyloidosis [18], only three patients had lysozyme amyloidosis. The reported median time from diagnosis of lysozyme amyloidosis to ESRD was at 10.6 years with all grafts functioning well without any evidence of amyloid recurrence. Liver transplantation has also been described in the literature; however, the procedure is not curative unlike TTR amyloidosis as cells other than the hepatocytes frequently produce lysozyme [13]. Immunotherapy-based options are in the early stages of evaluation for Alys [19].

This report highlights the need of awareness among physicians of this rare form of amyloidosis and, more importantly, that this is not limited to European ethnicities. Patients and family members who suffer from this disease require long-term follow-up. Genetic counseling and patient education about symptoms including the risk of bleeding is important, given the rarity of the disease and nonspecific symptomology. Multisite collaboration to identify patients and families with this rare disease to better understand the natural history and develop novel therapeutic approaches is critical.

## Figures and Tables

**Table 1:** Families reported in the literature with Alys.

Family	Mutation	Ethnicity	Dominant clinical features
1	W64R/TGG/CGG	French [1]	Nephropathy/Sicca syndrome
2	W64R/TGG/AGG	Italian [2]	Gastrointestinal
3	W64R/TGG/AGG	Italian [3]	Gastrointestinal
4	W64R/TGG/AGG	French [4]	Internal organ bleeding
5	I56T	English [5]	Nephropathy/easy bruisability
6	F57I	Italian [6]	Nephropathy
7	D67H	English [7]	Internal organ bleeding
8	D67H	English [8]	Nephropathy/GI bleeding
9	pTyr54Asn	Swedish [9]	Gastrointestinal/Sicca syndrome
10	I56T (our case)	South Asian	Gastrointestinal/nephropathy/internal organ bleeding
11	p.Leu102Ser	English with mixed lineage [10]	Nephropathy/neuropathy/gastrointestinal/cardiac
12	W64R/TGG/AGG	Italian [11]	Gastrointestinal
13	Mutation not specified; however, lysozyme C confirmed as amyloidogenic protein per LCMS	English [12]	Nephropathy/internal organ bleeding
14	D67H	English [13]	Internal organ bleeding
